# Supplementary material for: Warburg Effect, Glutamine, Succinate, Alanine, When Oxygen Matters
Source: Biology (Basel). 2021 Oct 4;10(10):1000. doi: 10.3390/biology10101000 (PMC8533123; doi:10.3390/biology10101000)
Supplement: Supplementary file 1 [file biology-10-01000-s001.zip › biology-1338347-supplementary.pdf]

biology

Supplementary information,

Warburg Effect, Glutamine, Succinate, Alanine, when Oxygen Matters

Frédéric Bouillaud [1]*, Noureddine Hammad[1], and Laurent Schwartz [2]

Figures S1 to S7

Glossary and abbreviations: S8.

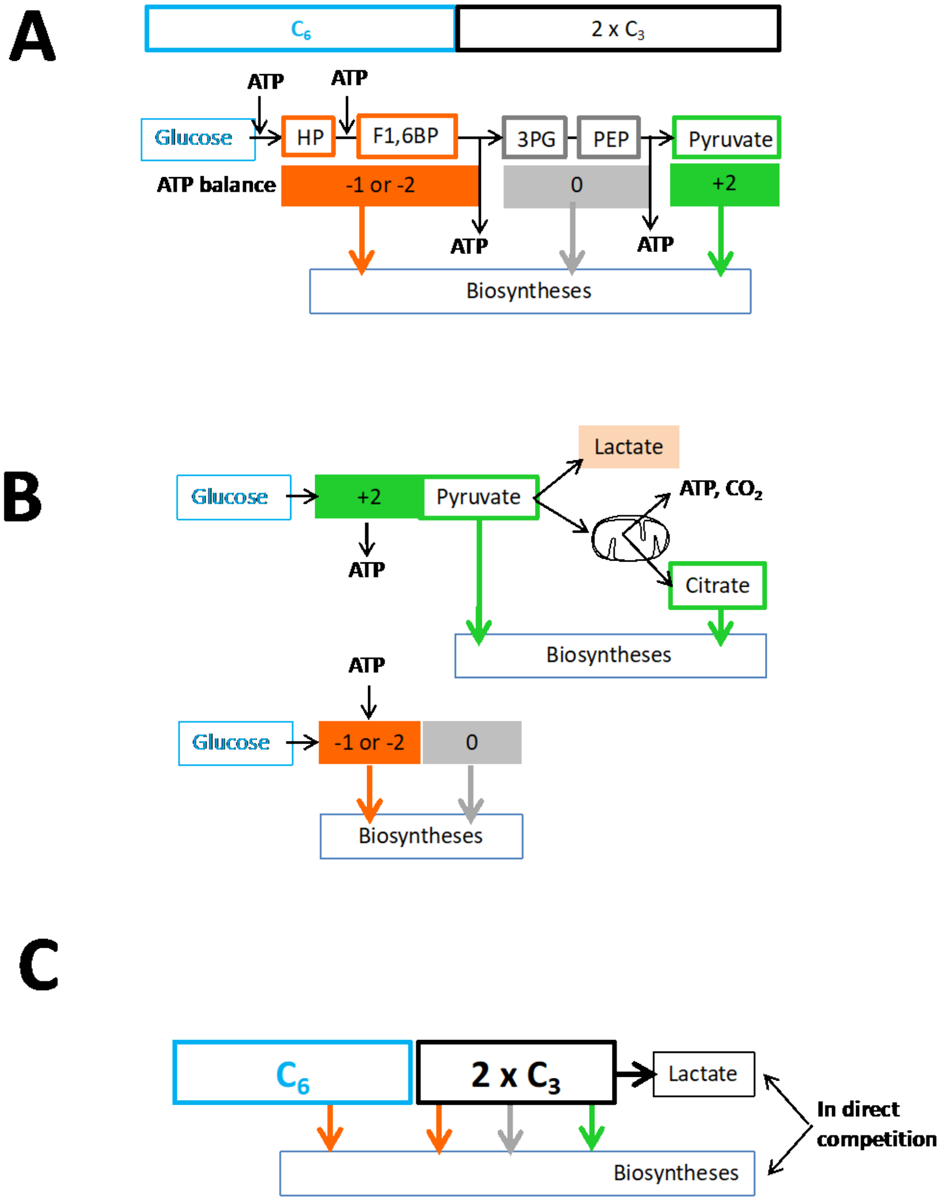

Figure S1: Warburg effect is a competitor for biosynthetic pathways.

Legend to figure S1: Warburg effect is a competitor for biosynthetic pathways.

A) Overview of the glycolytic pathway, Blue and black boxes on the top indicates the extent of reaction involving six carbon compounds (blue), and the reactions with 3 carbon intermediates (black, with two rounds per glucose). Below the glycolytic intermediates relevant with regard to ATP balance, are indicated. Abbreviations are: HP for hexose phosphates (glucose 6-phosphate and fructose 6-phosphate), F1,6BP fructose 1,6-biphosphate, 3PG 3phosphoglycerate, PEP phosphoenolpyruvate. The ATP balance is indicated below by colored boxes, in orange negative values (-1, -2) mean that one or two ATP have been used to activate sugars, in grey null balance two ATP have been obtained from the oxidation of two glyceraldehyde 3-phosphate, and green positive value (+2) net production of two ATPs after the last step of glycolysis (PEP to pyruvate). The downward arrows (with the same colors) illustrate that derivation of biosynthetic intermediates would withdraw compounds that, with the exception of pyruvate, result in a null or negative ATP balance.

B) The glycolysis contributes positively to cellular bioenergetics (net ATP production) if it proceeds up to pyruvate. The biosynthetic pathways then concern pyruvate fate which could be either oxidized (maximal ATP formation) engaged into biosynthesis, or transformed in lactate. Diversion of glycolytic intermediates upstream of pyruvate results either in a negative or null ATP balance and decreases pyruvate formation hence impacts negatively on lactate release. Color code is the same as in A.

C) Altogether lactate formation and biosynthetic pathways are in direct competition for the use of carbon atoms from glucose. Color code is the same as in A

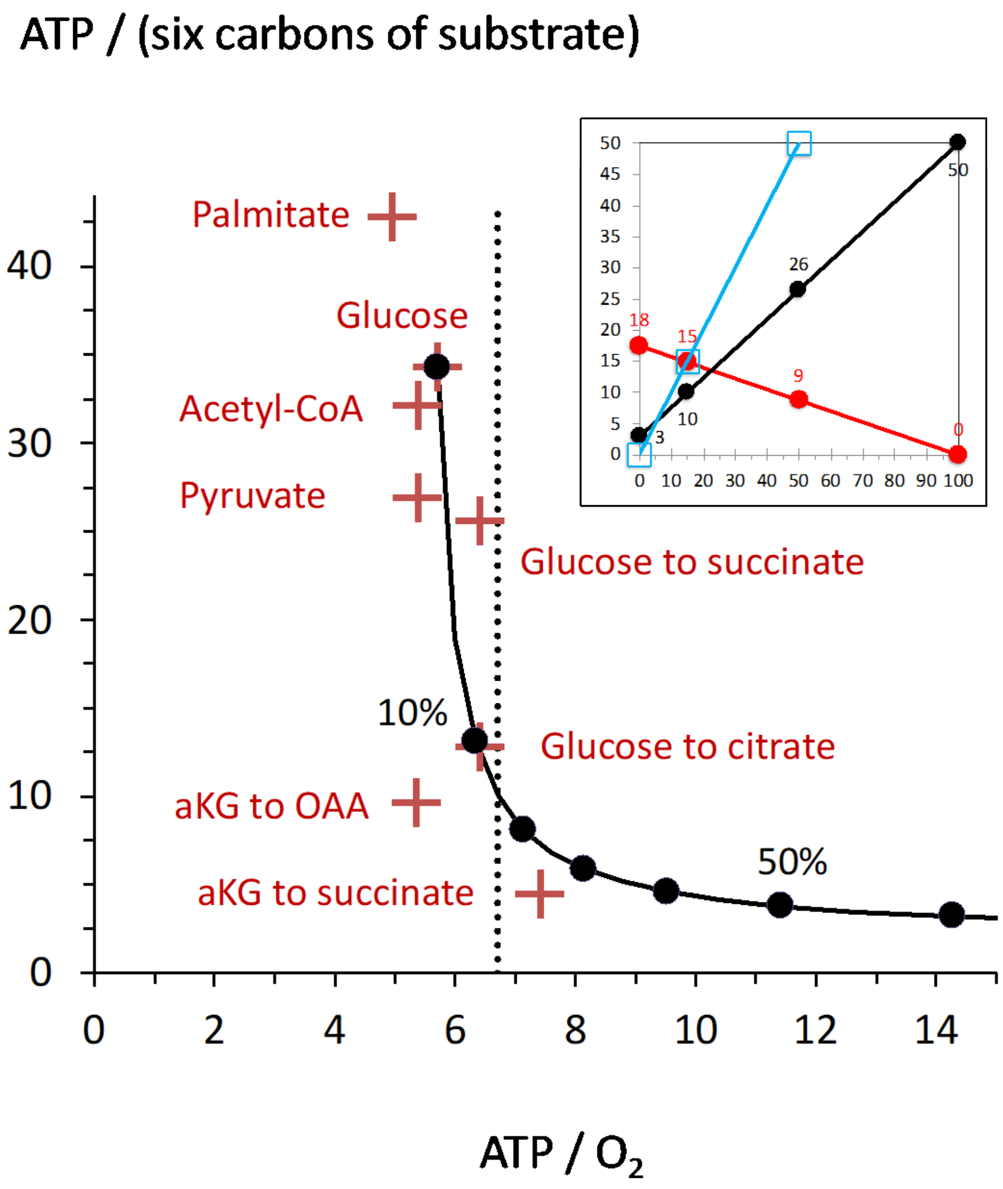

Figure S2: Large increase in ATP/O_2 ratio requires contribution of anaerobic ATP production.

Legend to figure S2: Large increase in ATP/O_2 ratio requires contribution of anaerobic ATP production.

The X axis represent the ATP yield according to O_2 use (ATP/O_2 ratio) and the Y axis the ATP yield for the mobilization of six carbons of substrates hence it is different from the value obtained with one substrate, for example two pyruvate are considered or 6/5 of a five carbons substrate such as alphaketoglutarate (aKG), OAA means oxaloacetic acid. When the final product is not specified (example Palmitate) crosses represent the value for full oxidation (all carbons oxidized to CO_2). Calculation of the ATP generation by the oxidative phosphorylation is made as follow: re-oxidation of NADH is supposed to be in all cases mediated by entry of electrons at the level of the *complex I* of the mitochondrial respiratory chain and to yield 2.7 ATP per oxygen atom (ATP/O_2=5.4). If the electrons come from a FAD/FMN intermediate, this includes Complex II of the mitochondrial respiratory chain,the yield is considered to be 1.6 ATP per oxygen atom (ATP/O_2=3.2).

Note that in the case of complete oxidation the yield per carbons of substrate and per CO_2 released are the same but their values are obviously different if partial oxidation is considered. For example, if the pathway from aKG to succinate is considered it mobilizes five carbons (aKG), releases one CO_2 and yields 3.7 ATP. The yield per six carbons of aKG is therefore 3.7×6/5= 4.4 (this figure). In contrast, with regard CO_2 the comparison with values for complete oxidation would require to consider six CO_2 hence six reactions and the value would be 3.7×6=22 hence closer to the values obtained with glucose or pyruvate.

The black curve starts from the values for full oxidation of glucose and represent the evolution as the contribution of lactic fermentation to ATP formation increases. Dots represent successive increases of 10% with 10% and 50% indicated. The vertical dotted line highlights the ATP/O_2 ratio of 6.7 that results from a 15% contribution of lactic fermentation to ATP formation, a situation in which the lactate release rate and the oxygen (O_2) consumption rate are equals (see inset). This allows comparison with the different oxidative pathways (crosses) and shows that only a conversion of aKG to succinate takes place with a higher ATP/O_2 (7.4).

Inset: Lactate release (blue) and consumption of oxygen (red) or glucose (black) as the contribution of lactate fermentation to the cellular ATP turnover rate increases. The generation of 100 ATP molecules is considered, X is the number of ATP generated by lactic fermentation the rest is supposed to come from glucose oxidation, then with X=0 values from the full oxidation of glucose into CO_2 are represented: no lactic acid generated (Y=0), approximately three glucose and 18 oxygen (O_2) molecules are used bythe oxidation pathway. The numbers of glucose and oxygen used are also indicated for two remarkable situations: lactate and oxygen flux are equal in intensity (15% lactic ATP) or lactic fermentation and oxidation contribute equally to ATP generation (50% lactic ATP). In the former case the glucose flux is 10 (three times increase) and the oxygen flux is 15 (ATP/O_2=100/15≈6.7), in the latter case the oxygen flux has a value of 8.8 and the lactate flux of 50 hence 5.7 times higher.

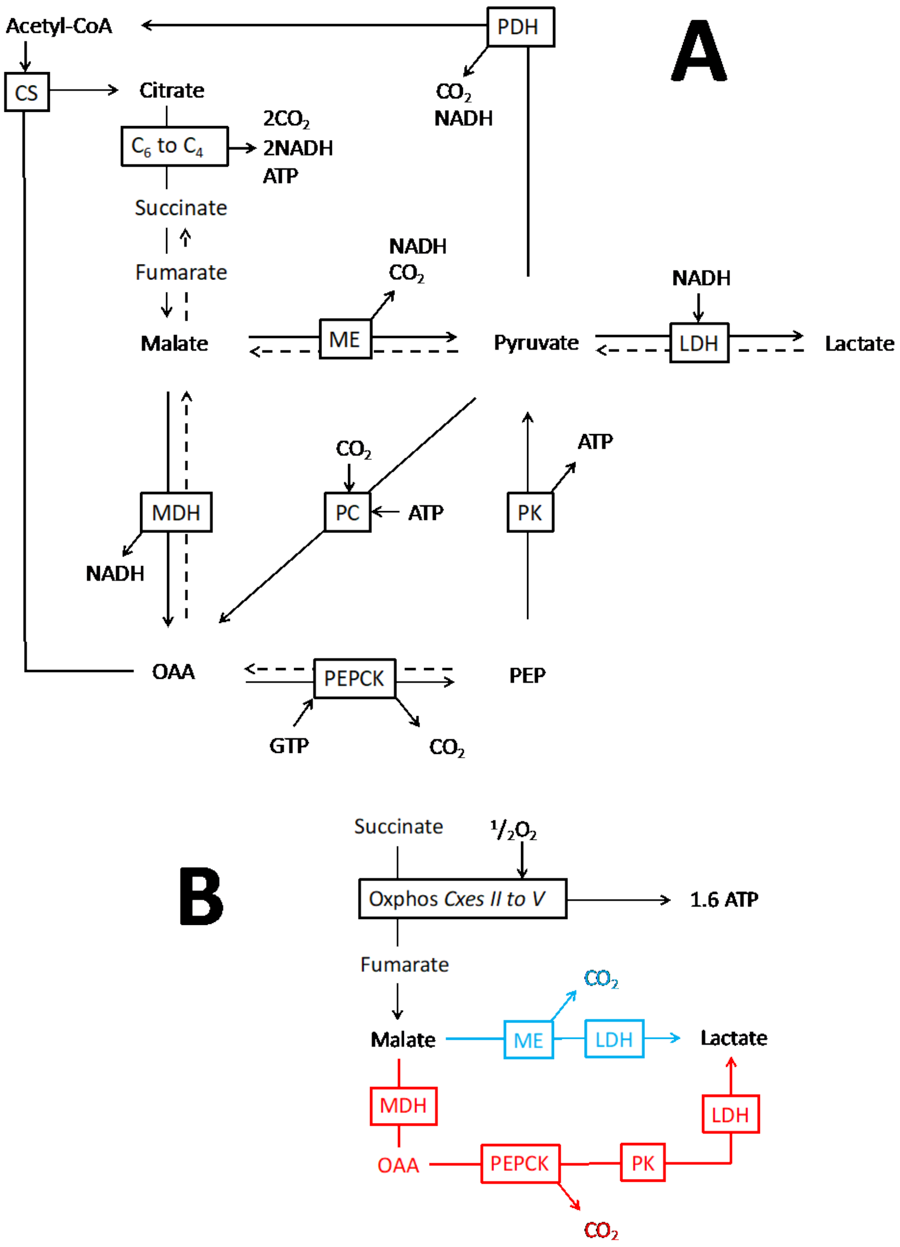

Figure S3: Metabolic network

A) Here are represented the relationships between the enzymes (PEPCK, PC, ME), metabolism of pyruvate (LDH, PDH, PK, PC, ME) and the TCA reactions from citrate to oxaloacetate (OAA). NADH, CO_2 or ATP use/release is shown only for one orientation (solid arrow), when the reaction is considered as reversible (dotted arrow) the result is exactly the opposite.

B) Oxidation of succinate in absence of complex I activity: Fumarate or malate are possible end products. However, from malate two pathways would lead to the formation of lactate: ME and LDH (blue) or MDH, PEPCK, PK and LDH (red), with regard to this pathway GTP and ATP are considered as equivalent. OAA and to a lesser extent malate are inhibitors of the *complex II*, lactate formation appears therefore as a likely option.

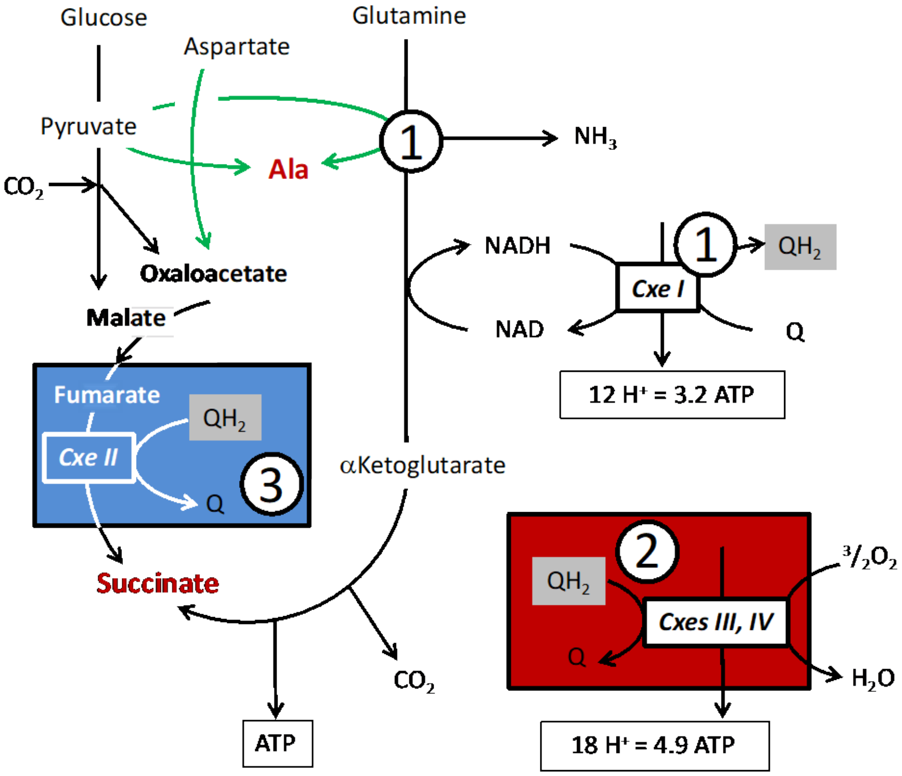

Figure S4: Glutamine to succinate reactions feed mitochondrial oxphos .

(1) A first set of reactions starts from glutamine and ends with succinate: Glutamine yields alphaketoglutarate (aKG) this may occur through transamination shown here with pyruvate from glucose releasing alanine (Ala) or through deamination of glutamate. Both pathways generate two NADH, the alanine pathway would release one ATP from glycolysis (not shown) and therefore results in better ATP/O_2 ratio. Alphaketoglutarate is an intermediate of the Krebs cycle that proceeds up to succinate. This short sequence of Krebs cycle releases one CO_2 one ATP/GTP (Succinyl CoA to succinate step) and reduces one NAD into NADH. Altogether three reduced coenzymes (NADH) are generated. Their reoxidation by the complex I of the mitochondrial respiratory chain results in the reduction of three quinones (QH_2). Re-oxidation of these quinones could proceed through two different ways:

(2) the respiration (red box) with complexes III and IV and use of oxygen, more details in figure S5.

(3) Under anaerobic conditions using a segment of Krebs cycle (blue box), more details in figure S6.

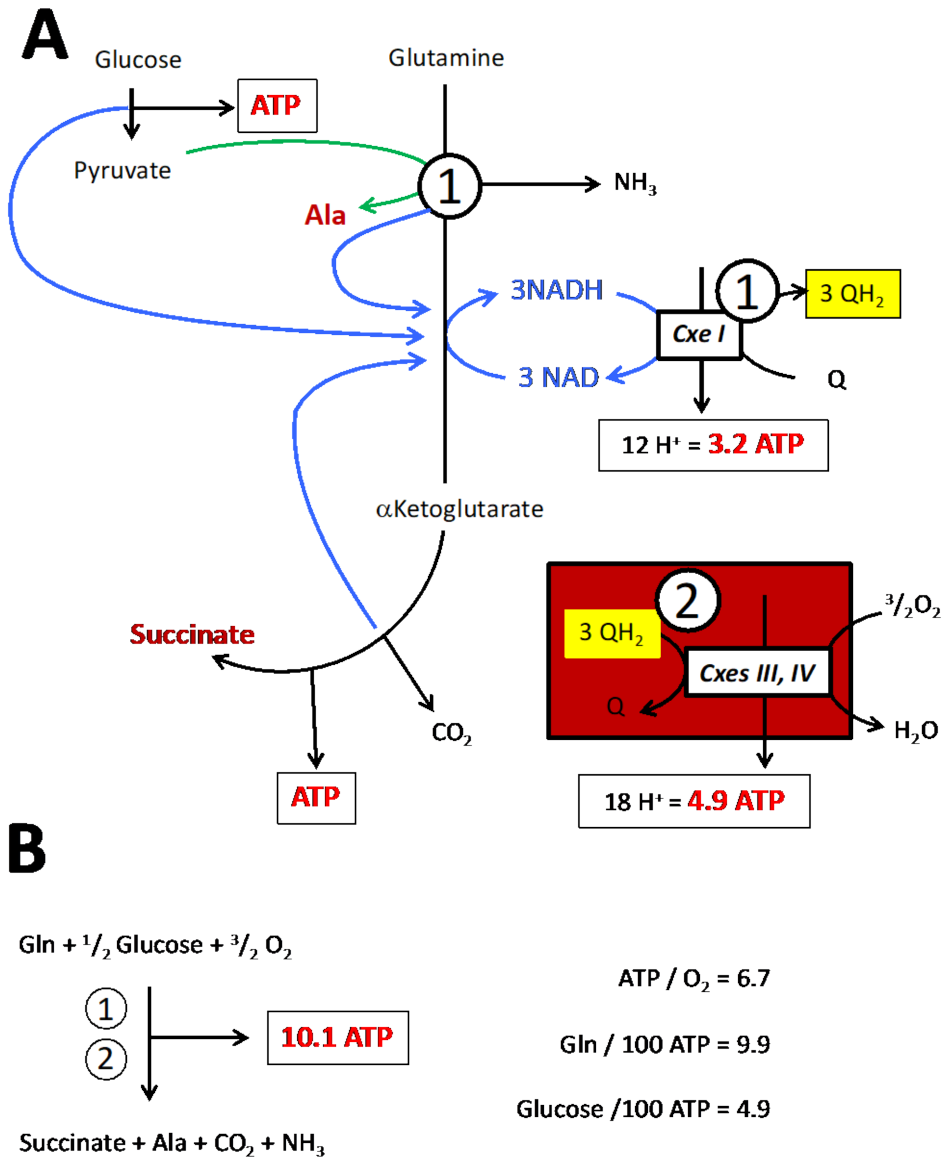

Figure S5: Glucose and glutamine to succinate under aerobic conditions.

A) (1) Glutamine and glucose yield alanine and alphaketoglutarate (aKG) that is oxidized to succinate. This releases two ATP and three NADH that via complex I reduce three quinones. (2) These quinones are re-oxidized by complexes III and IV (blue box) with use of oxygen. Mitochondrial complexes I, III and IV pump protons with the following relationships: four protons per complex I reaction two per complex III and four per complex IV, hence complex III and IV together yield six protons. These protons are considered as equivalent to ATP with a theoretical stoichiometry of 3.7 H^+ per ATP.

B) Reaction equation and ratios

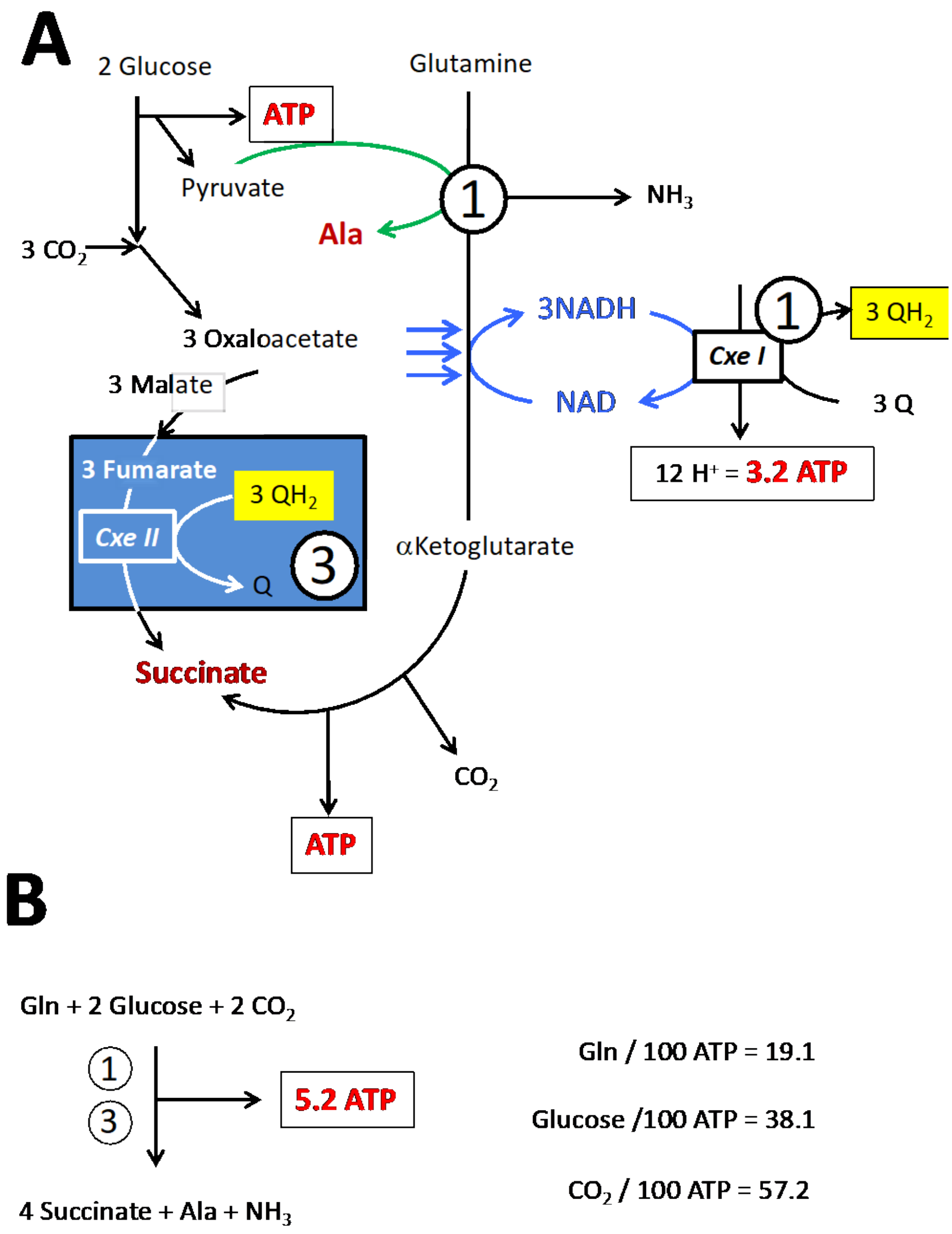

Figure S6: Glucose and glutamine to succinate under anaerobic conditions.

A) (1) one Glutamine and half a glucose yield alanine and alphaketoglutarate (aKG) that is oxidized to succinate. This releases two ATP and three NADH that via complex I reduce three quinones. (3) Reoxidation of QH_2 proceeds through the mitochondrial complex II working in reverse mode, replenishment in fumarate is obtained by the reactions of Krebs cycle from oxaloacetate to fumarate hence orientated towards reduction of the four carbons dicarboxylic acids. Feeding of this reversed Krebs cycle segment operates at the level of oxaloacetate or malate obtained by transamination of aspartate or carboxylation of pyruvate (see Figure A5). Succinate and alanine are the products of this anaerobic pathway and both were associated to adaptation/response to oxygen shortage. The mitochondrial complex I pumps protons that are equivalent to ATP molecules, the theoretical stoichiometrey 3.7H+ per ATP is used here.

B) Reaction equation and ratios

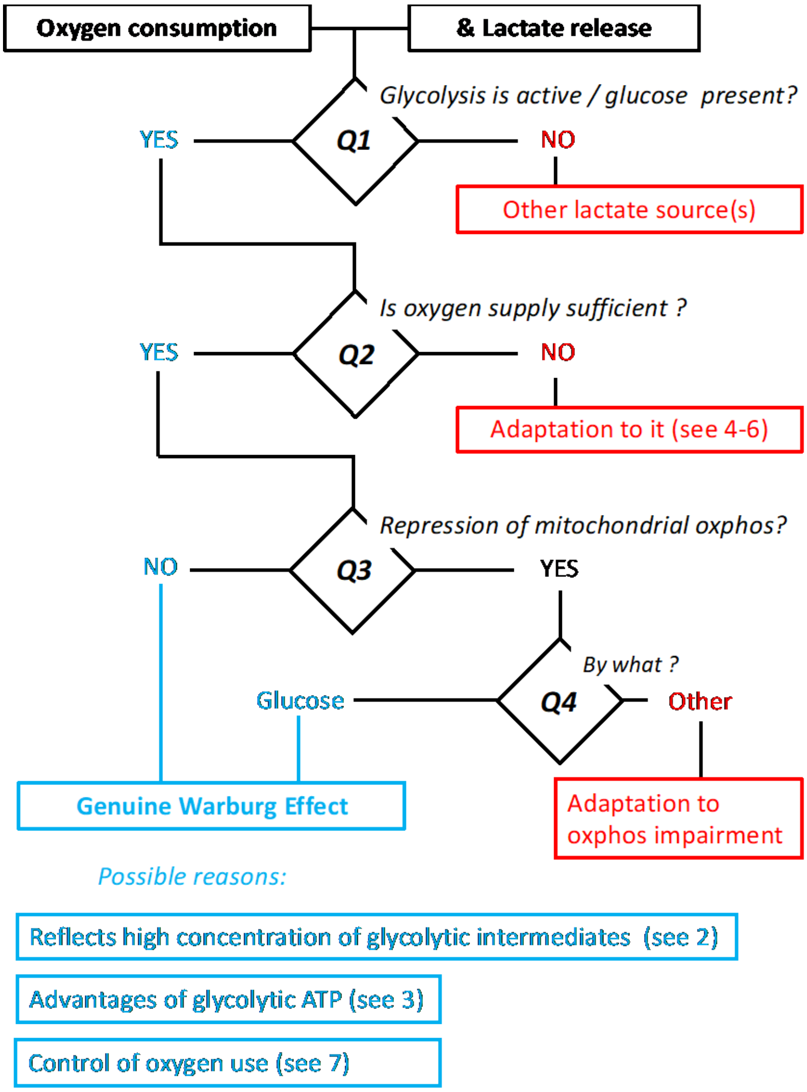

Figure S7: A decision flowchart to establish/or not the presence of a "genuine Warburg effect", *i. e.* the occurrence of aerobic glycolysis that is not imposed by oxphos impairment. Firstly, the components of the Warburg effect should be present: lactate release and respiration (oxygen consumption). The first question refers to the fact that Warburg effect requires a significant rate of glycolysis, this could be simplified to the presence of a significant (close to, or more than physiological) glucose concentration (response "yes" to Q1). Furthermore, the oxygen supply should authorize a 100% oxphos ATP generation (answer "yes" to Q2). While this is not easy to quantify, indirect evidence could be gathered if the oxygen consumption rate could be increased by addition of an uncoupler, a test easy to perform if cellular bioenergetics is probed by the "Seahorse approach" (https://www.agilent.com/en/product/cell-analysis). At this step a genuine Warburg effect is likely. However, because uncoupler probes for respiration and not for oxphos, confirmation would require that inhibition of oxphos could be excluded (answer "no" to Q3) or that this inhibition results from the action of glucose (Q4). The reasons for the presence of the Warburg effect as well as the alternative explanations (red boxes) are mentioned with references to the different chapters of the article.

Supplemental information S8: Glossary and abbreviations

Items are listed by alphabetical order of the abbreviations

aKG: alphaketoglutarate

ATP: Adenosine Triphosphate

Comments: the hydrolysis of the last "high energy phosphate bond" releases ADP (adenosine diphosphate) and phosphate (Pi). This reaction feeds the largest part of energy demanding cellular processes. Regeneration of ATP requires the inverse reaction: the phosphorylation of ADP. Few enzymatic steps do it as well as the mitochondrial oxidative phosphorylation (see below)

CS: citrate synthase

Comments: CS catalyzes the reaction below this reaction introduces the two carbons of the acetyl group to form citrate the first intermediate of the TCA cycle.

$$\text{Acetyl-CoA} + \text{oxaloacetate} \quad \rightarrow \quad \text{Citrate} + \text{CoA}$$

FAD: flavin adenine dinucleotide

FMN: flavin mononucleotide

Comments: both are redox intermediates or shuttles (see NAD, NADH below) and exchange two electrons and protons.

$$FAD + 2e^- + 2H^+ \quad \rightleftarrows \quad FADH_2$$

$$FMN + 2e^- + 2H^+ \quad \rightleftarrows \quad FMNH_2$$

With regard to oxidation in the respiratory chain, the conversion of the redox energy into ATP is expected to yield 1.6 ATP per $FADH_2$/$FMNH_2$ oxidized. This oxidation uses one atom of oxygen reduced into water. The ATP/O or ATP/O_2 ratio quantifies the yield of ATP generation with regard to oxygen with values of 1.6 or 3.2 respectively. Notably, these values are lower than that for oxidation of NADH and inclusion of FAD/FMN redox steps in an oxidative pathway impacts negatively on its global ATP/O_2 value.

Glycolysis ATP utilizing steps

1) Hexokinase (HK) catalyzes the first reaction of glycolysis the phosphorylation of a sugar with six carbons with hydrolysis of one ATP molecule.

$$\text{Glucose} + \text{ATP} \quad \rightarrow \quad \text{Glucose 6-phosphate} + \text{ADP}$$

2) Phosphofructokinase (PFK-1) catalyzes the phosphorylation of Fructose 6-phosphate into Fructose 1,6-biphosphate.

Glycolysis ATP generating steps

1) Glyceraldehyde 3-phosphate dehydrogenase (GAPDH), and phosphoglycerate kinase

Glyceraldehyde 3-phosphate + NAD + Pi ⇄ 1,3-Biphosphoglycerate + NADH

1,3-Biphosphoglycerate contains a high energy phosphate bond, the action of phosphoglycerate kinase is required to yield ATP.

1,3-Biphosphoglycerate + ADP ⇄ 3-Phosphoglycerate + ATP

The GAPDH reaction could therefore take place in absence of the phosphate acceptor ADP, this might deserve further consideration.

2) Pyruvate kinase (PK) is the last step of glycolysis, it catalyzes the reaction

Phosphoenolpyruvate + ADP → Pyruvate + ATP

HK: Hexokinase (see above: glycolysis ATP utilizing steps)

LDH : lactate dehydrogenase

Comments: LDH catalyzes the formation of lactate and NAD from pyruvate and NADH (see NAD, NADH below). This is the enzyme for lactic fermentation. The LDH would equally regenerate pyruvate by the opposite reaction depending on the redox status.

Pyruvate + NADH ⇄ Lactate + NAD

Actually, the ratio between lactate and pyruvate is considered as an index of the cytosolic redox state.

MDH: malate dehydrogenase

Comments: MDH catalyzes the reaction shown below, this is the last step of the TCA that regenerates oxaloacetate the acceptor for the reaction of citrate synthase (CS).

Malate + NAD ⇄ oxaloacetate + NADH

ME: Malic enzyme

Comments: ME catalyzes the reaction shown below, the reversibility of this reaction is documented.

Malate + NAD ⇄ Pyruvate + NADH + CO_2

NAD, NADH: Nicotinamide dinucleotide

Comments: NADH is the reduced form and NAD the oxidized form. The correct writing of the redox reaction is as follow:

$NAD^+ + 2e^- + 2H^+ \rightleftarrows NADH + H^+$

NAD is therefore a redox intermediate that allows exchange of two electrons between different enzymes/pathways. The term redox shuttle is used for this role. For example, NAD reduction by glycolysis is compensated by NADH oxidation by LDH (see above) or by mitochondrial respiration. The share between these two reactions determines the relative contribution of lactic fermentation and of mitochondrial oxidative phosphorylation to the cellular ATP turnover.

With regard to oxidation in the respiratory chain, the conversion of the redox energy into ATP is expected to yield 2.7 ATP per NADH oxidized (see ATP above). This oxidation uses one atom of oxygen reduced into water. Then the ATP/O ratio has a value of 2.7 or $ATP/O_2 = 5.4$.

OAA: oxaloacetate

Oxphos: oxidative phosphorylation

Comments: when eucaryotic cells are considered "oxphos" means implicitly "mitochondrial oxphos". Mitochondrial oxidative phosphorylation implies enzymatic complexes numbered from *I* to *V* (see figure 2). *complexes I-IV* are respiratory enzymes catalyzing successive steps for the electron transfer from reduced coenzyme (NADH, $FADH_2$ or $FMNH_2$) to oxygen. These *complexes I-IV* constitute, *sensu stricto*, the respiratory chain.

The functional organization of this respiratory chain (figure 2) shows that the reduction of quinone is a converging point for *complexes I and II*. In addition, other significant metabolic steps yield reduced $FADH_2$/$FMNH_2$, whose reoxidation depends on a quinone reducing enzyme, and therefore add further convergence of electrons to quinone.

The spontaneous redox reaction of respiration is associated to the proton pumping by *complexes I, III* and *IV*. It converts therefore the redox energy into an electrochemical gradient across the mitochondrial inner membrane (Mitchell's chemiosmotic theory). The conversion of energy from this proton gradient into ATP implies mitochondrial *complex V*, it requires also transporters to exchange ATP, ADP and Pi between mitochondria and the rest of the cell.

The stoichiometric relationship between electron transfer (two electrons per reaction) and proton pumping are considered to be four protons for *complex I*, two for *complex III* and four for *complex IV*. The *complex II* does not pump protons and yields the two electrons to complex III, this applies also to all reactions for reoxidation of $FADH_2$/$FMNH_2$. Similarly, a stoichiometric value is considered for the generation of ATP with 3.7 protons per ATP. This corresponds to 2.7 protons for the phosphorylating reaction (ADP+Pi → ATP) by *complex V* and one for the exchange of ATP against ADP+Pi between mitochondria and cytosol.

The ATP yield with regard to oxygen use is quantified by the ATP/O or ATP/O_2 ratios directly derived from the stoichiometric values above 10/3.7= 2.7 ATP if NADH is reoxidized by *complexes I, III, IV* and 6/3.7=1.6 for *complex II*, $FADH_2$/$FMNH_2$. These values are therefore theoretical, and maximal, because they assume a 100% yield (no proton leakage aside the *complex V*). This illustrates why oxphos and respiration are not synonymous, if respiration is uncoupled *complexes I-IV* consume oxygen but a large proton leak rate prevents the phosphorylation of ADP by *complex V*. Then respiration (O_2 consumption, CO_2 release) can be intense but oxphos would be absent. Alternatively, *complex V* inhibitors impair oxphos but cannot prevent respiration to take place at a reduced rate.

Determination of extracellular fluxes by the "Seahorse" methodology uses greatly these concepts and recourses intensively to different poisons that are troublemakers of the oxphos machinery.

PC: pyruvate carboxylase

Comments: PC catalyzes the reaction shown below, its reversibility is uncertain/excluded.

$$\text{Pyruvate} + CO_2 + \text{ATP} \rightarrow \text{oxaloacetate} + \text{ADP} + \text{Pi}$$

PDH: pyruvate dehydrogenase

Comments: PDH catalyzes the reaction shown below.

Pyruvate + CoA +NAD → Acetyl-CoA + CO_2 + NADH

PEP: Phosphoenolpyruvate

PEPCK: phosphoenolpyruvate carboxykinase

Comments: PEPCK catalyzes the reaction shown below, to a certain extend it might be reversible.

Oxaloacetate + GTP → phosphoenolpyruvate + GDP + CO_2

Phosphoenol pyruvate is a potential source of ATP via pyruvate kinase, (see Glycolysis ATP generating steps). ATP and GTP could be considered as mutually interconvertible through the action of the nucleoside-diphosphate kinase (NDPK). Then successive action of PECK and PKresults in production of pyruvate with release of CO_2.

PK: pyruvate kinase (see above: glycolysis ATP generating steps)

TCA: Tricarboxylic Acid cycle also known as Krebs cycle.

Comments: This is a sequence of reactions taking place in the mitochondrial matrix. The usual description starts from condensation of acetyl-CoA with oxaloacetate (OAA) to generate citrate. Then oxidation of the carbon atoms of citrate could proceed with two steps of carbon dioxide release accompanied by reduction of NADH and end with the formation of the first four carbon intermediate: succinate. This formation implies one step of substrate linked phosphorylation generating one ATP (red). In the subsequent steps succinate (COOH-CH_2-CH_2-COOH) is oxidized to generate oxaloacetate (COOH-CO- CH_2-COOH) that becomes available to initiate a new round of oxidation reactions. This oxidation path for the four carbons intermediates starts with succinate dehydrogenase (SDH), which is a FAD enzyme that is known also as the *complex II* (*Cx II*) of mitochondrial respiratory chain, it ends by the oxidation of malate (Mal) into OAA by malate dehydrogenase a NADH generating reaction. The reduced coenzymes formed are indicated by blue boxes with the theoretical factor (ATP/O ratio) for the conversion of the redox energy into ATP by oxidative phosphorylation.

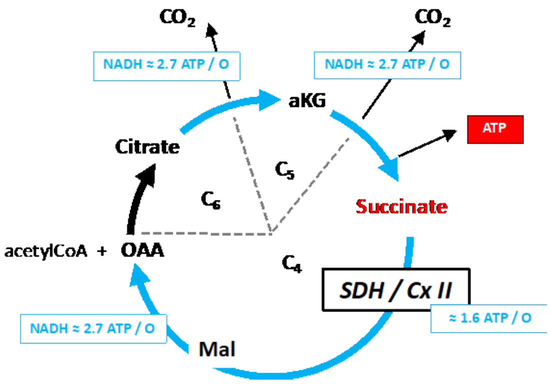

The TCA is then a major pathway for carbon oxidation and contributor to cellular ATP generation. The oxidation of one acetyl-CoA generates one ATP by substrate linked phosphorylation and (3×2.7)+1.6=9.7 by oxidative phosphorylation with the use of four oxygen atoms (two oxygen molecules O_2).

The TCA is also an important a branching point for a large number of biochemical intermediates (4-6 carbons organic acids). Then it implies a subset of TCA reactions with one intermediate as the entry and another one as the exit. Two terms are used to define the exchanges between TCA and the rest of the metabolic pathways: If metabolism feds the TCA with new intermediates, it is called anaplerosis. At the opposite, if the TCA feeds another metabolic pathway by losing intermediates it is called cataplerosis.

A simplification useful here is to consider that the first part of the TCA is essentially irreversible, and oriented in the oxidative orientation because of the condensation into citrate and the NADH/CO_2 releasing steps show strong negative ΔGs. This is indicated in the scheme by single clockwise (black/blue) arrows. In contrast, the C4 branch between succinate and OAA could be considered as reversible. The oxidative orientation (blue arrow) is necessary to regenerate OAA. At the opposite, it could proceed in the reductive orientation (red arrow in the scheme below) and for example consume OAA or malate to generate succinate. Then NADH is oxidized and *complex II* operates in the opposite direction with no oxygen consumption and no ATP formation.

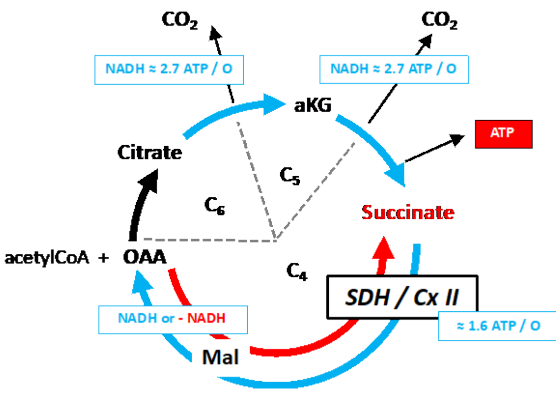

The figure below illustrates the relationships between glycolysis and TCA cycle with their impact on ATP generation by substrate linked phosphorylation (red) or oxidative phosphorylation indicated as the blue boxes showing the theoretical conversion of NADH/$FADH_2$ reoxidation into ATP. Completion of glycolysis releases two pyruvates, two NADH and two ATP (red box) per glucose. Then the fate pyruvate is the main issue.

- It could be oxidized by pyruvate dehydrogenase (PDH). Together with glycolysis this would lead to four NADH and release two CO_2 (remind that one glucose yields two pyruvates), then the TCA would proceed and terminate carbon oxidation (release of four CO_2) with further reduction of coenzymes and direct ATP formation.

- The pyruvate could be reduced into lactate by the lactate dehydrogenase with reoxidation of the two glycolytic NADH. The result is then the release of two lactates and two ATPs per glucose.

- The pyruvate feeds the reductive formation of succinate, then ATP and/or NADH are required to generate malate. Altogether this would cancel the glycolytic contribution to NADH/ATP formation it also implies assimilation of CO_2. How it takes place and why this may constitute a valuable pathway with regard to cellular ATP formation under conditions of oxygen restriction is explained in the text.

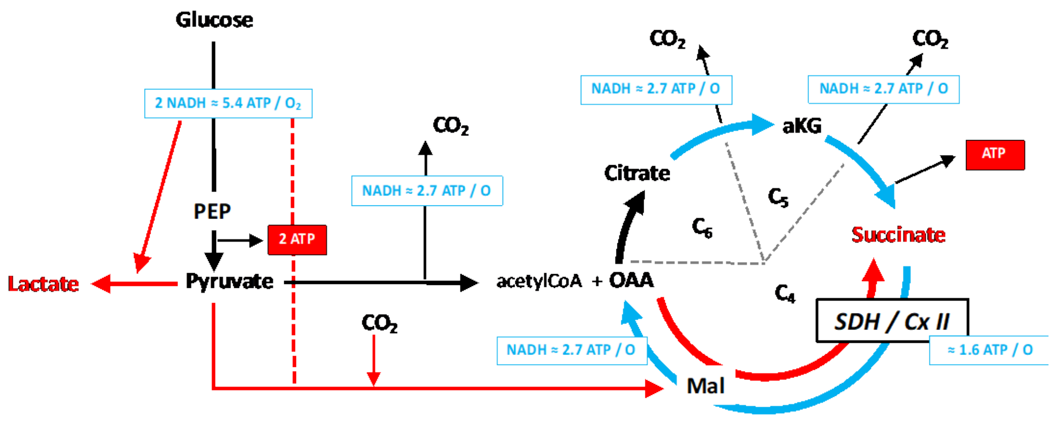
Glucose
2 NADH ≈ 5.4 ATP / O2
PEP
2 ATP
Lactate
Pyruvate
CO2
NADH ≈ 2.7 ATP / O
acetylCoA + OAA
CO2
NADH ≈ 2.7 ATP / O
Citrate
aKG
CO2
NADH ≈ 2.7 ATP / O
ATP
C6
C5
C4
Succinate
SDH / Cx II
≈ 1.6 ATP / O
NADH ≈ 2.7 ATP / O
Mal
CO2
